# The Role of Socialisation in the Taming and Management of Wild Dingoes by Australian Aboriginal People

**DOI:** 10.3390/ani12172285

**Published:** 2022-09-03

**Authors:** Adam Brumm, Loukas Koungoulos

**Affiliations:** 1 Australian Research Centre for Human Evolution, Griffith University, Nathan Campus, Brisbane, QLD 4111, Australia; 2 School of Humanities, University of Sydney, Sydney, NSW 2006, Australia

**Keywords:** tamed dingo, socialisation, Australian Aboriginal culture, domestication

## Abstract

**Simple Summary:**

The dingo (*Canis dingo*) is a wild-living canid endemic to mainland Australia; the descendent of an early lineage of dog introduced thousands of years ago to the continent, where it was isolated from further introductions of domestic canines until European colonisation began in 1788. Dingoes are notoriously difficult to maintain in captivity and owing to their predatory nature it is also known that they can pose a serious risk to children. Yet, written records and oral histories indicate that Aboriginal people in mainland Australia routinely practiced the rearing and keeping of dingoes in a tame state within their home communities and domestic spaces. This paper reviews historical and archaeological evidence for the management of wild and captive dingoes by Indigenous communities, revealing a substantial divide between the nature and outcomes of these interactions between historical/pre-contact Aboriginal societies and those in contemporary Australia. It is concluded that this special human-wild canid relationship has implications for the understanding of the domestication of dogs from wolves during the Late Pleistocene.

**Abstract:**

Historical sources and Indigenous oral traditions indicate that Australian Aboriginal people commonly reared and kept the wild-caught pups of dingoes (*C. dingo*) as tamed companion animals. A review of the available evidence suggests Indigenous communities employed an intense socialisation process that forged close personal bonds between humans and their tame dingoes from an early age. This was complemented by oral traditions which passed down awareness of the dangers to children posed by wild or unfamiliar dingoes, and which communicated the importance of treating dingoes with respect. Together, these practices resulted in what can be interpreted as substantially altered behaviours in tamed dingoes, which, despite their naturally high prey drive, were not considered a serious threat to children and were thus able to be maintained as companion animals in the long term. This relationship is of importance for understanding the original domestication of the dog, as it demonstrates a means by which careful and deliberate socialisation by foragers could both manage risks to children’s safety posed by keeping wild canids in the domestic realm and retain them well into reproductive maturity—both issues which have been highlighted as obstacles to the domestication of dogs from wolves.

## 1. Introduction

**Figure 1:**
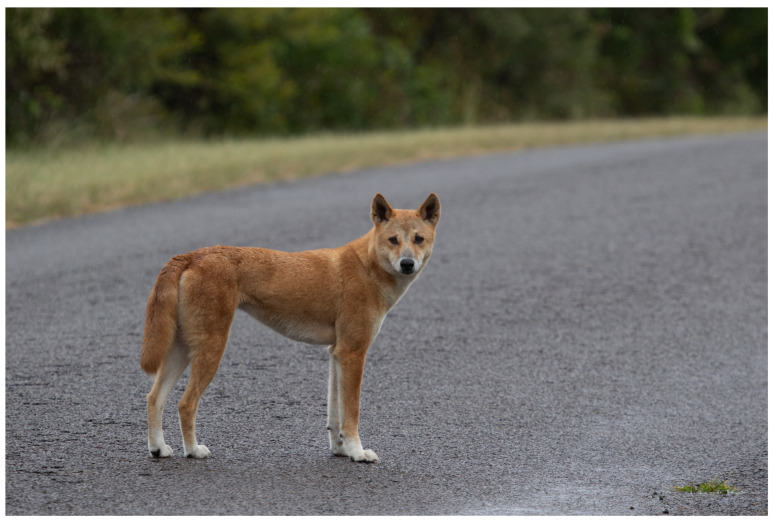
A dingo (*Canis dingo*). The photograph was taken in the Hunter Valley region of eastern New South Wales in 2022. Credit: Bradley Smith.

The Australian dingo (*Canis dingo* Meyer, 1793) is a medium-sized (15–16 kg) wild-living canid that was introduced to mainland Australia through human agency at some stage in late prehistory, and is now the largest mammalian predator on the continent [1,2,3] (Figure 1). Archaeological evidence suggests dingoes had been translocated to Australia by 3348–3081 calibrated radiocarbon years before present [4], although an earlier (and contentious) arrival time has been inferred on the basis of genetic studies [5]. Prior to the introduction of the dingo, there had been no canids in Australia. Scholarly debate continues over the dingo’s historical origin and its status upon arrival [6]; with one possible scenario being that dingoes descend from an offshoot of early domesticated dogs (*C. familiaris*) that reverted to a wild ancestral state in Australia [3,7]. The dingo is generally regarded as being intermediate between free-ranging wolves (*C. lupus*) and modern domestic dog breeds in terms of behavioural traits and reproductive biology [2].

At the time of the establishment of the British penal colony at Port Jackson (Sydney, Australia) in 1788—the first European settlement in Australia—dingoes were commonly seen together with local Aboriginal people; for example, residing in their camps, sharing their domiciles, travelling on foot and in watercraft with them, or otherwise in their company (e.g., [8,9,10,11]). This close human-canid relationship intrigued the British colonisers, with some remarking that dingoes were the only animals that Indigenous Australians had (apparently) “domesticated” ([10], p. 71). When colonists tried to rear dingo pups for themselves, however, they failed to produce canines whose behaviour resembled that of European domestic dogs ([12], p. 430). Instead, settler-reared dingoes were described as uncontrollable and irredeemably “savage” creatures that could not be entrusted around livestock owing to their strong predatory drive ([8], pp. 174–175). This was an early indication that there was something unique about the Aboriginal-dingo relationship.

As European settlement expanded into other areas of mainland Australia, it became apparent that Aboriginal people almost everywhere ubiquitously kept dingoes as “domestic dogs” [13]. In some communities, these animals were employed as hunting assistants for capturing prey [14]. However, the observed roles of these so-called “camp dingoes” varied from group to group, and for many Indigenous people their value as companions (“pets”) seems to have been foremost [15]. European colonisation resulted in profound and widespread disruptions to the customary lifeways of Aboriginal societies, including the breakdown of the traditional relationship between Indigenous people and dingoes [15]. Furthermore, introduced domestic canines were also rapidly adopted into Aboriginal communities, where they were widely valued both as hunting dogs and companion animals [14,16]. Consequently, by the 20th century anthropologists were only able to observe traditional practices of dingo-keeping in a few isolated areas [17,18,19,20,21].

In the 1960s, anthropologists and other scholars settled on the view that the dingoes seen living in Aboriginal camps during the early period of European colonisation were not domesticated in the conventional sense—that is, animals selectively bred under human control [22]. Rather, as a general rule camp dingoes seem to have been wild-caught canids that Aboriginal people took from dens in the Bush when they were very young pups, and hand-reared in their communities (e.g., [23,24]). It is broadly assumed that once these reared pups reached sexual maturity (roughly 12–24 months of age) they tended to disperse into the wild in order to reproduce [15] (for early references to this, see, e.g., [25], p. 196; [26], p. 4). Thus, the available evidence suggests that each new generation of camp dingoes comprised wild-born pups that were tamed and adopted by humans [3,15].

It was also recorded that Aboriginal people maintained conceptual distinctions between wild dingoes and tamed ones ([27], p. 190), as evidenced by the recurrence of separate terminologies for each throughout the continent, often with further subdivisions to designate juveniles and males/females of each group (e.g., [28], p. 161). One of the more detailed sources [29] provides insight into some of the reasoning behind such etymologies, in this instance among the Kaurna people of the Adelaide region. For the wild dingo, the general word for “dingo” *kadli* is given the prefix *warru*- meaning “without” or “outside”, and to describe dingoes in a tamed state, the prefix *wodli*- meaning “house” or “hut” [29]. For the Kaurna, at least, the terms for wild and tame dingoes therefore demarcate and define them, in a literal sense, by their relation to the human domestic space: those that live within it and those that live without it [29].

The broad consensus among present-day scholars is that despite the close relationship between Aboriginal people and dingoes the latter were generally free-roaming canids that lived independently of humans [3,15,30,31]. While some aspects of this standard story can be questioned [32,33,34], it does seem evident that where the Aboriginal–dingo relationship was documented it was based on the capture and (temporary) adoption of wild-born pups [15]. Apart from these aspects, much remains unknown about the nature of the earlier human–dingo relationship [32,33,34]. Of particular significance is that the interspecies companionship that was observed to have existed between Aboriginal people and dingoes seems to have no direct parallel in modern experience. Some non-Indigenous Australians keep genetically pure dingoes as pets in contemporary society (although the practice is illegal in some states and territories) [35,36]. However, it is widely recognised that dingoes are unconventional and highly demanding pets that are extremely difficult to train and socialise with humans [35,36]. They also lack most of the desirable traits of a standard household animal companion [35,36].

It is especially difficult to reconcile the Indigenous practice of keeping dingoes as social companions with the natural predatory behaviour of these wild canids. Dingoes are top-order predators with an innate hunting drive and the physical power to pull down and kill much larger prey (e.g., kangaroos) [1]. Furthermore, in recent decades wild dingoes that have become habituated to the presence of humans have been “responsible” for a spate of well-documented—though statistically rare—predatory attacks against non-Indigenous people, including two fatalities involving small children [37,38]. Given that Aboriginal people commonly took dingo pups from the wild and habituated them to live with humans, the question remains as to how they were able to cohabitate safely with a genetically-wild carnivore in their communities; especially given the risks these animals should have posed to babies and small children and other vulnerable members.

The aim of this paper is to address this question in detail—how did Aboriginal people manage to live harmoniously with a potentially dangerous wild predator in the domestic realm? Redressing this problem will contribute to the fragmentary knowledge of the special relationship between Aboriginal people and dingoes, a human-canid association often argued to provide a model for understanding how Late Pleistocene foragers in Eurasia domesticated the wild wolf ancestor of modern dogs [30,31,33,34,39]. In fact, the question posed here is also relevant to the study of the origins of dog domestication, given that the first generations of canids to reside with humans are likely to have been genetically-wild wolves or their slightly modified descendants [40,41,42,43]. 

After reviewing the evidence for the nature of the human–dingo relationship, it is proposed that the Aboriginal practice of hand-rearing wild-born dingo pups involved a process of intensive socialisation that affected the behaviour of camp dingoes in a way that is not presently well-understood. This possibly included suppressing the human-socialised dingo’s natural predatory drive around humans in general, and babies and small children in particular.

## 2. History of Dingo Attacks in Non-Indigenous Australia

Wild dingoes have long been maligned in European-Australian society as sly and ravening pest animals, a widespread attitude that can be traced to the beginnings of a sheep production industry in the late 18th century [44]. Dingoes were (and are) despised by pastoralists for their relentless (and supposedly cruel and wanton [45]) predatory attacks on domestic livestock, especially sheep [1,46]. Anecdotal accounts exist of captive/pet dingoes attacking their owners or other people in the earlier period of Australian history and in modern times (e.g., [47,48,49] and see below). There were also reports of “doggers” (dingo trappers) being mauled by wild dingoes that they had cornered or wounded (e.g., [50]). Until recently, however, the wild-living canids had not been perceived within modern Australian society as representing a direct physical threat to humans, unless they had been provoked or goaded into a defensive attack [51].

**Figure 2:**
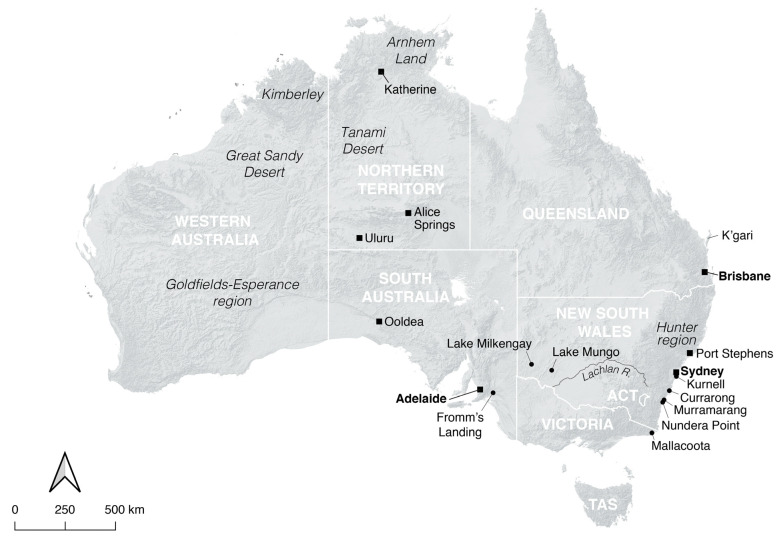
Map of Australia showing the places mentioned in the text. ACT = Australian Capital Territory. TAS = Tasmania. Base map prepared by Kimberlee Newman.

Indeed, this popular conception was a key issue in the wrongful conviction of Lindy Chamberlain, a non-Indigenous woman whose nine-week-old daughter, Azaria, disappeared from the family’s tent at an Uluru campsite (Figure 2) in August 1980, and who was subsequently imprisoned for her murder [52,53]. Mrs. Chamberlain professed her innocence, claiming that a wild dingo had snatched Azaria from the tent and carried her off into the Bush; but her testimony was widely disbelieved, and she was ultimately convicted and received a life sentence (quashed in 1986) [52,53]. There are a number of reasons why her account of how her baby had died was held to be false by so many (for multifarious analyses of the Chamberlain affair, see chapters in [54]). Central among them, however, was the entrenched belief held in the Australian community at that time that free-ranging dingoes simply do not attack humans without provocation [51,52,53].

**Figure 3:**
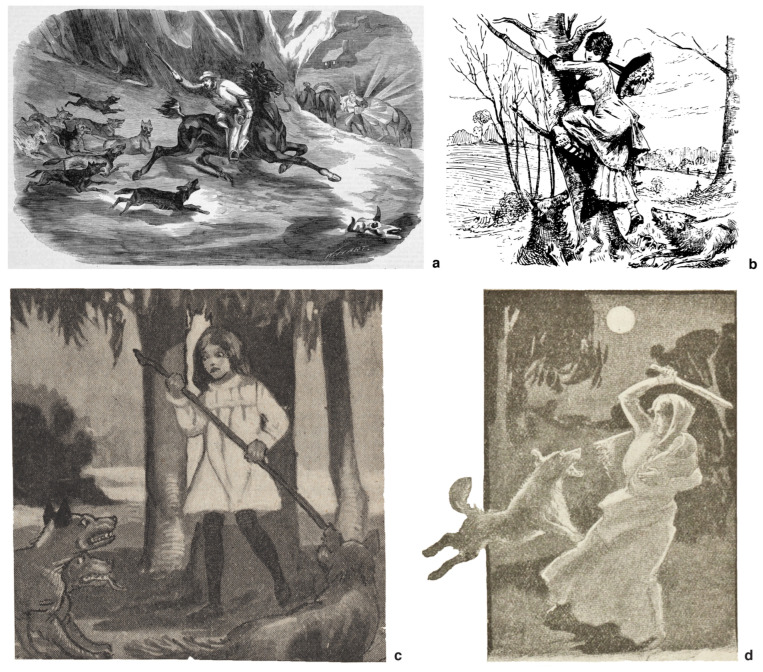
Early depictions of dingoes attacking European settlers. (**a**) Dingoes pursuing a man on horseback. This is a portrayal of an incident that is supposed to have taken place in the Para River region (South Australia) in the mid-19th century [55] (**b**) A pair of dingoes chasing a woman up a tree [56]. Published in 1884 (redrawn from the original using the Adobe Illustrator Live Trace tool [setting: Default]), this is an illustration of an event that is purported to have taken place in 1884 in Queensland [51]; (**c**) dingoes circling a lost child [57]; (**d**) a dingo attacking a woman carrying an infant [58]. The latter two artworks are from fictional stories. Sources: (**a**): ‘The Dingo Or Native Dog’ (Walter Hart, 27 July 1866), State Library Victoria; (**b**): Title: ‘Tree’d by dingoes: a pretty girl on the Dawson River, Queensland, takes refuge from the attentions of native dogs of too much taste’ [56]; (**c**,**d**): National Library of Australia.

It is little known, however, that up until the early 20th century it was commonly believed in Australia that dingoes *did* opportunistically prey on humans, especially unsupervised children [51]. Newspaper articles and oral traditions from the latter half of the 19th century in particular describe wild dingoes killing and feeding upon children who were lost in the Bush, but also inebriated adults, riders who were thrown from their mounts and injured, and “swaggies” (itinerant Bush labourers) who travelled through rural areas alone and on foot (Figure 3) [51]. It is difficult to be certain whether these early anecdotal accounts of dingo attacks refer to empirical events or if they are myths with roots in older European wolf lore, but at least some of the historical reports fit with details of modern dingo attacks [51]. In any case, the colonial-era narrative that dingoes were opportunistic predators that occasionally preyed on people appears to have been erased from the public imagination during the interwar period [51].

**Figure 4:**
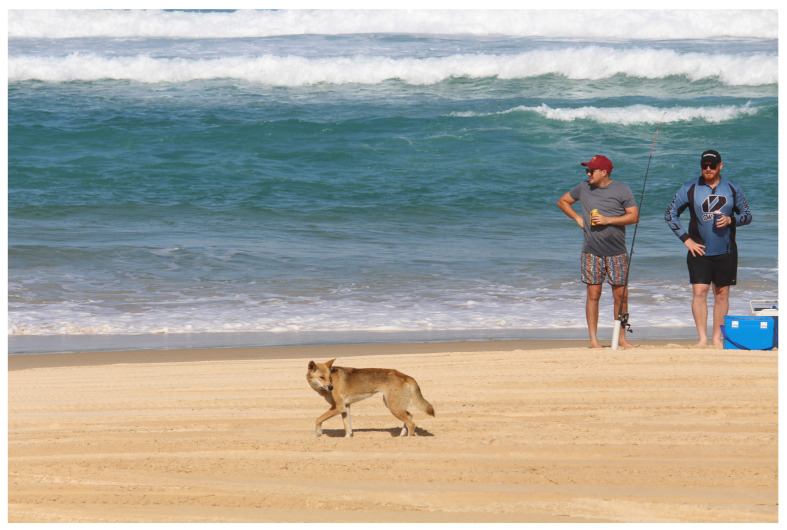
A dingo on K’gari (Fraser Island). This free-ranging individual is habituated to the presence of humans, as are many others on this popular holiday destination where people regularly camp, fish, and engage in other recreational activities in wild dingo habitat. Credit: Frankie Dixon (Unsplash).

In modern Australia, a pronounced cultural shift in attitudes towards dingoes has only occurred since the 1990s in light of the various negative human-dingo incidents that have occurred on K’gari (Fraser Island), where the resident population of free-ranging dingoes (~160–260 individuals) is a major tourism drawcard [59,60] (Figure 4). Some incidents are interpreted as dingoes biting or nipping people in a “playful” manner [37,38,60]. On other occasions, however, dingoes have approached or ambushed young children at campsites, beaches, townships, or other places, seized the youngsters in their powerful jaws, and tried to drag them away [61,62,63,64,65]. Moreover, in a well-publicised incident that took place in 2019, a dingo apparently snatched a 14-month-old child from a camper trailer and pulled him into the scrub [66]. In each instance, a parent or guardian intervened, leaving the rescued child injured but alive. In 2001, however, a nine-year-old boy was tragically mauled to death by a pair of juvenile (~22-month-old) dingoes that began following him while he was walking with a male friend of similar age [37]. Most negative encounters between dingoes and people occur during the annual breeding (March-May) and whelping (June-August) seasons, when dingoes are more aggressive and under stress to feed young pups, respectively [38].

As a result of the recent events on K’gari it is now generally accepted in Australia that some dingoes potentially will, under certain circumstances, attack and kill humans, especially children, in order to consume them as food—indeed, the Chamberlains were finally exonerated by a 2012 royal commission on the basis of the K’gari incidents [37]. It should be noted, however, that predatory dingo attacks are extremely uncommon and that the vast majority of interactions between dingoes and humans are benign [37,38]. The conventional wisdom is that the truly wild dingoes of K’gari—the ones that live in the more remote parts of the island and hence have no (or very limited) contact with people—are afraid of humans, ardently avoid them, and therefore pose no direct threat. It is generally thought to be the case that some K’gari dingoes only became “problem dingoes” in the early 1990s when cases emerged of habituated dingoes attacking or threatening humans [59]. On the other hand, it is not always clear what had motivated individual dingoes to bite or threaten humans [37,38]. Furthermore, contrary to popular belief there are, in fact, historical accounts of wild dingoes attacking people on K’gari long before the advent of the modern tourism industry; indeed, the first report of an attack on a European is from 1836 ([67], p. 66; see [51] for a discussion of other early incidents). In other words, it may not be the case that it is only in recent decades that some wild dingo populations have posed a legitimate danger to humans [51].

## 3. Aboriginal People and Wild Dingo Predation

There is little reliable information available about cases of dingoes attacking Aboriginal people during the early period of contact with Europeans or in the more recent past where a traditionally-oriented foraging lifestyle continued in remote areas—at least one early European chronicler explicitly stated that he had ‘never heard [of an Aboriginal person] allude to [dingoes] as being dangerous either to themselves or their children’ ([23], p. 177). The coroner who presided over the first coronial inquest (1981) into the disappearance of Azaria Chamberlain was specifically interested in ascertaining whether dingoes had been known to prey on Aboriginal babies and small children; a question with obvious bearing on the plausibility of the Chamberlain defence [52]. The same issue was also raised during the 1986 royal commission into the Chamberlain convictions (the “Morling Inquiry”) [68]. At these proceedings, Lionel Perron—father of then-Northern Territory attorney-general, Marshall Perron—testified that he had been privy to such an attack while working as an engineer for a project in the Great Sandy Desert in 1961:

On an occasion when his party was camped near a group of nomadic aborigines a baby from the group, about twelve months old, was carried off by a semi-domesticated dingo. Mr Perron’s party recovered the partly eaten remains of the baby.

([68], pp. 280–281)

A newspaper article published at this time reported that Lionel Perron had observed an Aboriginal man from the camp trying to spear a ‘pet’ dingo: ‘When asked why, the Aborigine had replied that the dog had eaten a [child]. Mr. Perron said he shot the dingo but after opening it found no remains of the child. However, he was shown the child’s limbs, vertebrae and skull’ ([69], p. 7). This story is scant on detail and probably unverifiable, as are the handful of other anecdotal accounts of Aboriginal people being attacked by dingoes that materialised at the time of the Chamberlain affair [69,70]. The incident as reported by Perron also appears to have involved a socialised dingo “pet” rather than a solely wild, free-ranging animal.

The rarity of references to Aboriginal people older than babies or small children being attacked by wild dingoes could simply be a result of the patchy nature of the ethnohistorical record. However, it also seems likely to at least partly reflect the fact that some Aboriginal groups hunted dingoes for food [33,34], and hence wild dingoes in these areas would probably have been too fearful of humans—their main predators—to come within their vicinity. It is also likely that the circumstances under which people have been attacked by wild dingoes on present-day K’gari (but also in other parts of the country) would not have arisen as frequently, or at all, in traditional Aboriginal contexts.

A key problem on K’gari is obviously that wild dingoes are now a popular tourist attraction, as already noted [37]. Most of the humans the free-ranging dingoes encounter are recreational campers or other short-term visitors. Instead of keeping away from the animals and actively discouraging them from interacting closely with humans, it is common for visitors (and some local residents) to deliberately approach dingoes, hand-feed or lure them with food, or else passively tolerate their presence (e.g., campers allowing dingoes to feed on their rubbish) [37]. The agency responsible for dingo management and conservation on K’gari (Queensland Parks and Wildlife Service [QPWS]) disseminates a plethora of multimedia information on “dingo safety” rules to the public, exhorting visitors to: keep children close at all times; never walk or jog alone; and, if confronted by a dingo, to stand confidently and loudly call for help, maintain continual eye contact with the animal, and never try to run away (i.e., do not act like the dingo’s prey) [71]. It is clear that not all visitors heed these warnings, however [37,38].

With regard to the experiences of Aboriginal people, it seems reasonable to argue that humans who lived in the open in unfenced encampments (see below) and hunted and foraged in the Bush on foot would have had a considerable incentive to avoid doing things that led to wild dingoes becoming habituated to their presence—a potentially dangerous situation. It is also likely that in ‘traditional’ times all members of Aboriginal communities would have been taught from an early age how to behave or respond appropriately should they encounter a wild dingo up close—the Indigenous cultural equivalent of modern western “dingo safety” rules [71]. It is also evident that oral traditions (myths and stories) communicated knowledge about the dangers posed by free-ranging dingoes and how to avoid them ([72] and see below).

Wild dingoes might also have been wary of predating on Aboriginal people because of the risk of personal harm this entailed. Like most predators, dingoes tend to avoid prey that can severely injure or kill them, such as healthy adult cattle and buffalo ([1], p. 113). Dingoes on present-day K’gari have bitten and mauled numerous young non-Indigenous adults, not just unaccompanied children [37]. However, these people were typically on their own and on foot, unarmed, and not uncommonly in some kind of further position of vulnerability (e.g., inebriated) [51]. Aboriginal men and women, on the other hand, even if foraging or hunting in the Bush alone rather than in groups (which was the norm; see below), are likely to have been carrying material culture objects that could be used to swiftly kill or incapacitate a dingo. This would have included spears, boomerangs, or clubs in the case of men, while women routinely carried offensive implements like hafted stone axes and digging “sticks” (stout hardwood rods that doubled as fighting clubs ([73], p. 65; [74], p. 153). Of greatest danger to a dingo would have been any projectile that could kill at a distance; especially spears, which were often propelled with spearthrowers for greater accuracy and enhanced range, and increased force at closer distances [75]. Ethnohistorical images also suggest that Aboriginal boys typically learned how to use weapons of the hunt and war from an early age, with toys soon giving way to real armaments; and girls probably would have commonly had digging sticks with them [73]. Hence, older children were less likely to have been in danger of dingo predation compared with youngsters of equivalent age on modern-day K’gari.

In summary, it seems reasonable to assume that Aboriginal people would have known about the dangers of wild dingo predation and how not to place themselves (and their children) in situations or to generate conditions (i.e., wild dingo habituation) that could have made them more vulnerable to it [72]. But of course contingencies could have arisen in which it was not possible to heed cultural warnings. Moreover, some people might simply have made lapses of judgment that placed themselves or others in life-threatening situations.

In addition, while it is probably true that wild dingoes rarely attacked Indigenous adults or older children of either sex because of their propensity to defend themselves with lethal aggression, it is also evident that dingoes are intelligent and opportunistic predators that could have used the advantage of numbers or exploited other vulnerabilities to countervail such risks. For example, a correspondent to the *Northern Territory Times*, writing in 1928, claimed to know of a case in which an Aboriginal woman was set upon by a dingo while she was alone and carrying a baby, and thus at a major disadvantage [76]. She managed to fight the animal off with her digging stick, but was so badly mauled that she died several days later [76]. Another story related by an Aboriginal man (“Doolgan”) in 1886 described how he and his young nephew were waylaid by a group of wild dingoes when out hunting in the MacDonnell Ranges, near Alice Springs [77]. Although Doolgan had a hunting spear with him, he had put it down when he saw the dingoes so that he could place his nephew safely out of reach in a tree. He then threw rocks at the dingoes in an effort to drive them away. This may have been standard protocol for discouraging and/or scaring off dingoes that were too close for comfort. If so, however, it did not work on this occasion; instead, the animals came directly for Doolgan, forcing him to climb the tree without his spear. There Doolgan and the child stayed for the night while the dingoes waited watchfully nearby. Community elders later confirmed his story after they observed the dingoes’ tracks around the tree [77]. Notably, this account implies the dingoes had been fighting over a female in oestrus and thus that the incident took place during the breeding season when adults are more aggressive [38]. There are similar stories from the colonial period of settlers escaping dingo attacks by climbing trees [51] (Figure 3). A 23-year-old male backpacker was also “treed” by dingoes on K’gari in 2012 [59].

In any case, smaller Indigenous children were defenceless, and, if unaccompanied by older people, no doubt could have fallen prey to a predatory attack by a wild dingo, including in their home communities. With regards to the latter, there was no “typical” Aboriginal camp, and camp form and Indigenous architecture were more diverse than is popularly believed [78]. However, ethnohistorical accounts imply that most people lived in unfenced encampments consisting of clusters of impermanent dwellings; these were usually windbreaks or small bark huts, the former being only partly walled or enclosed, while other less substantial types of shelters included “lean-tos” [78]. The style of open living inherent to these camps, and their minimalist domestic structures [78], may have exposed younger residents to wild dingo predation. It would have been risky for a dingo to enter an occupied camp given the probability it would be killed if detected or tracked to its lair and dispatched. According to Daisy Bates, however, in the Ooldea region of inland South Australia wild dingoes were sometimes able to infiltrate camps and kill tamed dingoes before the startled residents could reach for their weapons [79]. Pastoralist Robert Dawson was also present in Aboriginal camps in the late 1820s that were subjected to nocturnal raids by wild dingoes in the Hunter region of New South Wales [23]. On one occasion, Dawson was awakened when dingoes entered camp late at night to steal some leftover kangaroo meat ([23], p. 178). The camp dogs confronted the interlopers, but were forced to retreat after a violent fracas; the rest of the camp slept through the bedlam [23]. At another time wild dingoes carried off a kangaroo carcass with such remarkable stealth that camp residents attributed the theft to a *debbil-debbil* (Aboriginal pidgin English word meaning malignant spirit) ([23], p. 162). In addition, Daisy Bates recorded the following testimony from the Ooldea desert community:

Dingoes howled on the sandhills all night through, and sometimes came in to the siding and killed the settlers’ goats and fowls: the natives told me that before the days of the white man, they had been known to slink in to the breakwind shelters at Uldilgabbi and attack the babies.

([80], p. 177)

These stories would seem to imply that infants or toddlers in camps might have been vulnerable to a wild dingo incursion, especially one conducted in the early hours or at other times when most people in the community would have been sleeping.

It is worth noting that modern attacks by dingo-like canids that are closely associated with Aboriginal people have been documented. Large numbers of canids of varying descriptions are a characteristic feature of many remote European-style Indigenous settlements (although these are places of permanent habitation, they are often known colloquially as “camps”) [19,81,82,83,84,85]. These “camp dogs” comprise both mixed-breed domestic dogs (including dingo cross-breeds) that cohabitate with community members, and free-roaming canids that maintain a loose association with people and are regarded by them as “family”. Notably, shortly after Azaria Chamberlain’s disappearance, one of the police investigators on the case claimed in a media interview that in the mid-1970s ‘camp dogs attacked and devoured a new-born child’ at Papunya, an Indigenous community located close to Alice Springs ([70], p. 6). Moreover, in 2022 a three-year-old Indigenous boy went missing from another Aboriginal settlement near Alice Springs, Hermannsburg, and was found in the Bush the following morning with critical injuries deemed to have been inflicted by local camp dogs, according to media reports [86]. 

## 4. Behaviour of Modern Captive Dingoes

**Figure 5:**
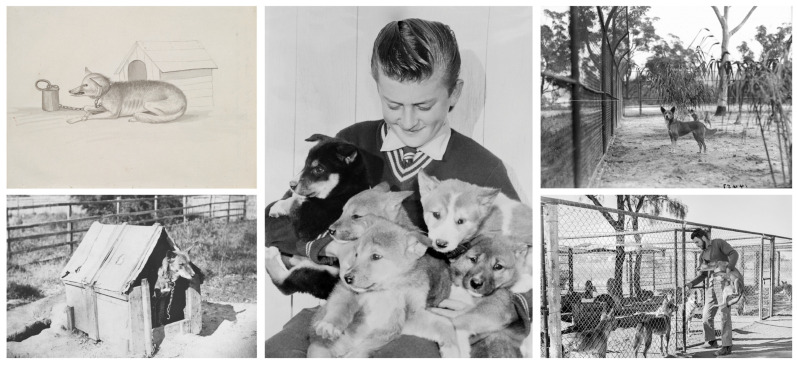
Captive dingoes and dingo pets kept by non-Indigenous Australians, with a focus on the typical restrictions on movement and high-walled enclosures deemed necessary for the keeping of adult dingoes. (**Top left**): A captive dingo *c*.1797, presumably obtained by an early European colonist in Sydney (**middle**): A young boy holds a number of dingo pups he was raising as a pet on his family’s farm in Queensland, 1965; (**top right**): Dingoes kept in captivity at Taronga Zoo, Sydney, 1930; (**bottom right**): Dingoes and officer at the Commonwealth Scientific and Industrial Research Organisation (CSIRO) captive dingo colony in Alice Springs, Northern Territory, 1967; (**bottom left**): Captive dingo kept in kennel at the Acclimatisation Society’s Garden, Brisbane, 1885. Sources: (**top left**): ‘A wild Dog or Dingo of N. S. Wales’ (Richard Pulteney, 1797): PXA 678/1, Mitchell Library, State Library of New South Wales; (**middle**): ‘Children’s Newsletter No 29—Ivan Rohlf, a 12 year old schoolboy in Queensland, with five dingo pups he was keeping as pets on his parents’ dairy farm’ (R. Nicol, 1965): National Archives of Australia, A1200 L52197; (**top right**): ‘Taronga Park Zoo. Dingo (1930): State Archives & Records Authority of New South Wales Government Printing Office 1-2227; (**bottom right**): ‘Research–Zoology–CSIRO experimental officer measures a dingo in NT dingo research’ (Australian News and Information Bureau, 1967): National Archives of Australia, A1200 L64195; (**top left**): ‘Captive dingo at the Acclatisation [sic] Society’s Garden, Brisbane’ (Francis Hall, 1885): State Library of Queensland.

Since the earliest period of British settlement in mainland Australia non-Indigenous people have endeavoured to keep wild dingoes as pets (Figure 5). Some early Sydney colonists, for example, acquired dingo pups and reared them, and some were even sent back to England as exotic gifts ([8], p. 174). The few written accounts left by British settlers of raising young dingoes for use as hunting “dogs”/pets invariably emphasised their fierce demeanour, irrepressible hunting instinct, and generally unruly behaviour. For example, the first governor of the colony of New South Wales, Arthur Phillip, noted that his pet dingo ‘is of a very savage nature, and not likely to change in this particular … it is scarcely to be expected that this elegant animal will ever become familiar’ ([8], pp. 174–175). Another 18th century colonial governor, John Hunter, recorded similar experiences:

Of those [native] dogs we have had many which were taken when young, but never could cure them of their natural ferocity … I had one which was a little puppy when caught, but, not-withstanding I took much pains to correct and cure it of its savageness, I found it took every opportunity … to snap off the head of a fowl, or worry a pig, and would do it in defiance of correction … I believe it to be impossible to cure that savageness, which all I have seen seem to possess.

([10], p. 67)

Of pet dingoes, lieutenant-governor David Collins simply wrote: ‘They have an invincible predilection for poultry, which the severest beatings could never repress’ ([11], p. 567).

Over the course of Australia’s European history many others have experimented unpropitiously with keeping wild-caught dingo pups as pets [87] (Figure 5). For instance, some colonial-era shepherds supposedly raised wild dingo pups to alleviate the boredom of their solitary existence [88]. Professional dingo trappers were also known to rear dingo pups to keep as decoy animals [89] and/or family pets [90]. Furthermore, it seems to have been common at one stage for pastoralists and other rural folk to adopt young wild-caught dingoes for use as working dogs and station pets [87]. Some breeders also retained captive dingoes for back-crossing with livestock herding dogs [87,88].

Among the early stories of dingo ownership is the recurring narrative that tame dingoes were mercurial companions that could be affectionate and “loyal” (i.e., like domestic dogs) but were also obstreperous and unpredictable; for example, they were supposedly prone to attacking their owners without warning [48,91]. It was therefore widely believed that pet dingoes were ‘never to be trusted’ [92]. Some early accounts are more positive in their assessments of the young tamed dingo’s character and suitability as a pet [93,94,95]. Even the most amiable of companion dingoes, however, usually ran away when they began manifesting traits of sexual maturity; or else they were abandoned or killed, as by then they had become “too wild” to keep as family pets. As one authority noted: ‘Sadly, the story was unfailingly the same—at adulthood their dingo was shot because it was a nuisance and an incorrigible hunter of anything that moved’ ([87], p. 4). Ward’s account encapsulates the travails of many early dingo-owners:

In the days when they were kept as pets about the stations it was never safe to leave them unwatched. They would do immense mischief to any articles they could destroy, such as curtains and household linen, books and papers, and everything that was tearable; and perhaps the owner would see his pet meekly blinking on some soft mat or skin, stretched in the graceful attitude of rest as if it had been calmly sleeping for the past hour, to find, when he passed on to the side of the house, some dozen or more of his choice breeds of fowls and ducks with their heads nipped off. It is small wonder that the dingo has ceased to be a favourite throughout Australia.

([91], p. 201)

**Figure 6:**
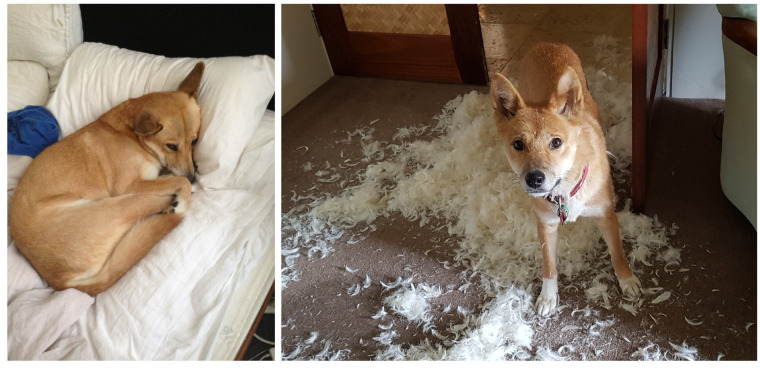
Dingoes kept as household pets in present-day suburban Australia. The dingo at right has just destroyed the owner’s pillow (image credit: Mel Browning). The photograph of the dingo on the couch is courtesy of Desiree Hemberger.

Modern experience confirms that keeping dingoes as household companion animals is a seriously challenging pursuit [35,36,87] (Figure 6). Surveys of modern dingo owners reveal an array of undesirable personality traits or behaviours displayed by pet dingoes [35,36,96,97]. These range from extreme independence—in this regard they are often likened more to cats than dogs by their owners/carers—to being highly fearful of novel stimuli and experiences ([36], p. 266). Lack of training focus is a common complaint, with pet dingoes consistently described as being ‘less attentive, reliable, biddable, trainable and obedient than [domestic] dogs’ ([36], p. 266). One seasoned dog trainer who spent decades working closely with companion dingoes concluded that: ‘It takes super-human patience and control to pursue the almost impossible dream—a well-trained Dingo whose behaviour is even remotely reliable’ ([35], p. 76). Dingoes are also notorious escape artists, howl constantly, are distrustful of strangers, and often highly destructive to property [35] (Figure 6); although so are many breed dogs. Owing to their high prey drive dingoes may also pose a risk to the physical safety of small children and to other pets and livestock if left unrestricted ([87], p. 101).

In short, dingoes are widely considered to be extremely difficult to manage as household companions in the contemporary western context [35,36,96,97]. The uncommon practice of dingo ownership has given rise to a small but impassioned network of devotees who keep these canids as exotic pets in urban areas of modern Australia [36]. However, well-meaning dingo owners often find that they are unable to cope with the challenge of what some describe as a full-time job, relinquishing the adult animals when they are still young ([35], p. 54)—many are euthanised as they are so difficult to rehome [35,36]. Notably, the behavioural challenges posed by contemporary captive dingoes persist after generations of breeding in captivity, in settings such as zoos and sanctuaries, despite other signs of captivity-induced phenotypic change in such populations ([98], p. 372).

## 5. Rearing and Socialisation of Camp Dingoes by Aboriginal People

The traditional model of the life cycle of the camp dingo is drawn from historical observations of Aboriginal communities from approximately the mid-19th to early 20th centuries (e.g., [25,99,100,101]), and partly based on the testimony of Aboriginal informants who had already ceased to raise wild-caught dingoes as companion animals but retained living memories of the practice [22,82,84,102]. Historically, this followed a consistent pattern: young pups were acquired, prior to the age of weaning, from litters born to wild-living dingoes. Available evidence suggests the pre-weaned pups were typically taken from dingo dens formed in caves, hollow logs, animal burrows, and other holes or subterranean cavities (e.g., [25], p. 195). Notably, among the Pitjantjatjara (Uluru area), and probably other groups in the region, den-raiding was an annual winter-time event linked to astronomical cues and entailed a community-wide endeavour ([103], p. 374). Pup-collection often occurred in order to acquire dingoes for human consumption, but even in these instances, and otherwise, some members of the litter were kept and taken home to be raised [15]. Although carefully provisioned and doted on in their puppyhood, these dingoes were weaned off close human caretaking as they aged into adolescence. With little nutrition freely available in the camp, and a strong urge to disperse and reproduce, tame dingoes departed human society soon after becoming sexually mature, dispersing back into the Bush in order to live and breed independently in the wild [15]; although it is possible that in some times and places there were individuals that either did not stray far from the camps or indeed essentially remained within them throughout their lives [32,33,34]. This section outlines the ways in which Aboriginal people managed tame dingoes, with particular attention to their efforts to socialise them to domestic life and thus produce acceptable behavioural dispositions.

### 5.1. Nurturing and Wet-Nursing

The wet-nursing of young dingoes by Aboriginal women, a relatively widely-observed phenomenon prior to the early 20th century ([99], p. 76; [104]; [105], p. 352; [106], p. 65; [107], p. 347; [108], p. 54; [109], p. 241; [110], p. 88; [111]; [112], p. 726), was interpreted by most of its observers as a physiological stimulus to prevent further pregnancy by recent mothers [33,34]. However, this was also a deliberate and crucial first step of the process of socialising camp-dwelling dingoes. As pups were collected prior to their weaning off milk by their mothers, their nutritional demands at this stage in life were subsequently met by human nursing.

There is ample ethnographic evidence for cross-species wet-nursing of young captive wild animals in small-scale human societies, including foragers ([41,113] and references therein). It would seem that no modern scientific experimental program has attempted to replicate this extreme (from a modern western standpoint) form of providing nutrition to hand-reared wild canid pups—or domestic dogs for that matter—and so the possible psychological effects of wet-nursing on the juvenile animal are essentially unknown. At any rate, constant handling by women during nursing and otherwise during this formative period would, under most circumstances, have led to the young dingoes being imprinted upon by humans; imprinting forms the social attachments between individual animals and humans that are essential to domestication and management [114,115].

It is important at this stage to also acknowledge that Aboriginal infants and children were typically present during dingo-nursing—sometimes being nursed at the same time—and otherwise were constantly exposed to one another’s presence (i.e., scent, noises, movements, and physical contact) [106,116]. Indeed, Aboriginal people in the Ooldea region reportedly would not seek to obtain a wild dingo pup to tame unless a baby had recently been born in camp [79]. Further, accounts suggest that tame dingoes roamed unrestrained around camp; they were, however, sometimes bound or tethered to prevent them from following men on their hunting expeditions, as they were regarded as a nuisance (e.g., [24]). They also slept with their adoptive family in huts, windbreaks, or other domestic spaces; in fact, on cold nights dingoes were apparently highly prized as “living blankets” (e.g., [117], p. 113). In addition, James Dawson wrote the following about Victorian Aboriginal groups: ‘When the family is travelling, the youngest child under two years old is carried on the mother’s back beneath her rug, occasionally in company with a young dingo’ ([118], p. 39). Similarly, according to a southeastern Queensland settler, when on the move: ‘The younger married women will usually have a child either in a pouch behind, or else astride their shoulders but the ratio of puppies to [small children] will usually be five to one’ ([119], p. 339).

It is also worth noting that according to the Ngadju people of the Goldfields-Esperance region (southern Western Australia) in former times smoke was used as part of the process of taming wild-caught dingo pups: in their words, the intentional exposure of pups to fire-smoke was believed to ‘take the wildness out’ of them ([120], p. 13). Such intentionality in taking measures to induce and instil ideal behavioural traits in reared camp dingoes is scarcely acknowledged in scholarly perspectives on the matter.

### 5.2. Rough-Handling

Also under-appreciated, on the other hand, is the phenomenon of rough-handling of young dingoes and later domestic dogs by Aboriginal people, and especially by young children, which is often remarked upon in the historical literature ([82], p. 289; [83,85]; see [33] for further discussion). For example, anthropologist Donald Thomson noted that in eastern Arnhem Land: ‘The aborigines generally handled the [tame dingo] puppies by one leg, by the ears, or even by the tail’ ([21], p. 18). Tonkinson observed similar treatment among the Martu people of the Great Sandy Desert region: ‘Tame dingoes are incredibly patient, allowing children to perpetrate all sorts of indignities on them without protest’ ([74], p. 48).

Musharbash expands on this, reporting that Warlpiri children’s striking, dragging, throwing, pinching, and imprisoning of young dogs was not only allowed but encouraged by parents or guardians, though if enacted upon another child this behaviour would result in immediate interference and rebuke by adults [85]. Rough-handling by children could, and often did, result in fatality: amongst some contemporary Western Australian Aboriginal communities, for instance, this form of human-canid interaction accounted for approximately 20% of pre-weaned pup mortality ([83], p. 251). However, it is claimed that the surviving dogs had instilled in them a ‘healthy respect’ for Warlpiri infants for the rest of their lives ([85], p. 97). One of Musharbash’s Warlpiri (Tanami Desert, Northern Territory) informants, a female Elder, credited this seemingly cruel socialisation process with an appreciable outcome in which camp dogs did not attack or injure children or adults, and, as a result, Warlpiri people held no fear of dogs [85]. According to Warlpiri beliefs, the rigours of rough-handling establish an understanding in camp dogs that humans, including infants, hold a superior position of physical power (i.e., they are ‘stronger’) ([85], p. 97).

The use of rough physical “play” to establish hierarchical positions of superiority and subordination between people and dingoes is perhaps in some way analogous to wild dingoes’ use of aggressive-dominant-submissive interactions to establish their own social pecking orders [121]. These behaviours and the proper reciprocal actions are taught to dingo pups by parents and older relatives, as established by experiments with pups raised in isolation [122,123]. As such, the possibility that with rough-handling Aboriginal people established themselves in the role of “superior” dingo can be entertained. However, it should be noted that domesticated dogs are widely recognised as inherently more submissive or deferential to humans than dingoes and other wild canids are—presumably the outcome of a long history of selective breeding for tameness behaviour and sociability [114]. Hence, techniques of this manner may have been more effective for the later domestic dogs of Aboriginal camps than with the dingoes which preceded them.

In modern western dog-training culture, punishment of juvenile domestic canids (i.e., hitting or other pain-based stimuli) is seen as counter-productive, as it is thought to commonly result in the dog developing abnormal social behaviours as an adult, especially increased reactivity and/or human-directed aggression (e.g., [124,125] and references therein). However, there have been no studies of the relative merits of rough-handling versus non-rough-handling methods of rearing domestic dogs in the context of contemporary Indigenous Australian communities. Moreover, some traits that can emerge as an outcome of punishment-based training (which can arguably be equated to rough-handling in the Aboriginal context)—such as “clingy” behaviour oriented towards owners [125]—might not have been viewed within Aboriginal culture as being undesirable if manifested by camp dingoes. At this stage, it is only possible to highlight the Aboriginal testimony for subjecting camp dogs (and possibly captive dingoes) to a prolonged phase of “cruelling” at the hands of children, and to note that the particular developmental-psychological processes are not well-understood.

Importantly, the Aboriginal socialisation process may have also surmounted the issue of dingoes associating humans with food handouts, losing the fear of people and subsequently behaving aggressively towards them in efforts to obtain food. Such food-oriented habituation is widely identified as the cause of negative interactions and/or injurious dingo encounters on K’gari (and has resulted in laws against feeding dingoes there) [37]. There can be little doubt that dingoes would have made this association with Aboriginal camps and their inhabitants, given the availability of food, which was provisioned, or otherwise available by scavenging or stealing. It is, therefore, possible to again observe intentionality in Aboriginal actions towards their young dingoes/dogs to produce behavioural ideals that successfully managed the dangers posed by these canids to vulnerable members of the community. Although Kolig ([126], p. 94) reports that other native animals captured and given as “pets” to children (see also [127]) were also treated in a similar manner and essentially ‘played to death’ ([74], p. 48), the taxa in question (large macropods) seem to have otherwise invariably been eventually eaten by people or the camp dogs ([82], p. 288). Hence, the expected trajectory of their lives was quite different from that of the tame dingo.

### 5.3. Corrective Behaviour

Socialisation practices applied to adult dingoes are, by the limited nature of their residence in camp, less well-demonstrated in historical and ethnographic literature. However, it is widely understood that as they aged into adolescence and early adulthood, irritating behaviours such as begging/pestering for food, thieving of food, and fighting amongst each other for food, were discouraged and punished with physical blows or other reprimands [17,82,83,128]. Concerning the latter, there are indications that some corrective behaviours took a form that was in dingo “terms”. For example, on one occasion in Arnhem Land, Donald Thomson was censured by his hosts for striking a camp dingo pup that had snapped at him [21]. The advice he received from onlookers was that he should have bitten the ear of the errant pup instead: ‘“Pay back”, they said–that is, the approved form of correction was to pay it in its own coin’ ([21], p. 17).

The few references to tame dingoes attacking people mostly coincide with the fewer references to Indigenous Australians disposing of (grown) tame dingoes, an act otherwise considered almost anathema [33,34]. According to traditional exegesis the Wardaman people (near Katherine, Northern Territory) used a plant-based poison for this purpose ([129], p. 176); and men of the Walmajarri people (Kimberley) would spear and eat tame dingoes or dogs that bit children ([130], p. 35). On a similar note, according to oral tradition the Butchulla would spear and eat any dingoes that acted aggressively towards people [131]. The wild/tame status of these K’gari dingoes is unclear, but even if they were the former, such actions potentially would have selected against aggressiveness in the dingoes that ultimately formed the basis of the Butchulla camp-dwelling populations. The QPWS’s policy of selectively culling “problem dingoes” that bite or persistently threaten people is similar, in essence, to this traditional Butchulla management practice [60].

## 6. Oral Traditions and Cultural “Law” Informing Dingo Management

Dingoes (or “dogs” more generally) are widely and prominently represented in Aboriginal spiritual traditions, myths, lore, and cosmology related to the Dreaming (Dreamtime)—the creationary epoch—where they often feature as important customary “law”-givers and as the companions of major mythical figures and Ancestral Beings [15,126]. However, dingoes also appear frequently as dangerous figures or forces: ravenous predators, in the form of shape-shifting beings, as assassins sent by enemies, and as hunting “dogs” in the employ or otherwise under the command of evil beings [72,83,132]. In these roles, dingoes are presented as destructive agents which attack, injure, and even kill and consume other important figures and “culture heroes” in Dreaming mythologies, often resulting in the formation of certain noteworthy landscape features or celestial arrangements.

Comprising a particularly widespread mythological motif is the giant dingo (or dingoes) who do the bidding of malevolent figures, usually old women and/or cannibals, and on their behalf attack and kill other Dreaming figures. The myths and stories they are contained within usually explain the origins of various bird and lizard species, and many appear to share a common origin. They are found throughout much of eastern Australia, with examples recorded from northern Queensland [133], northern New South Wales ([134], p. 24; [135], p. 110), western ([136], p. 66) and southern South Australia ([137]; [138], pp. 149–150); similar figures and events transpire in stories from the southeast Queensland ([100], p. 128) and northwestern Australia ([126], p. 102; [139]). Particular attention is drawn to this group of traditions because the victims of the mythical dingoes’ depredations are attacked from a vulnerable position. They are frequently children, and typically ones that were left alone by their parents who have gone out foraging. Other victims are vulnerable by virtue of being old and blind, and yet others are victimised because they live alone, have recklessly strayed from the group whilst travelling or foraging, or have been deceived into doing so by the dingoes’ master(s). This state of vulnerability, away from the care of family and community, makes them easy prey for the sudden, surprise attacks of the dingo.

It is also of relevance to this discussion to note the existence of oral traditions in which people and communities who mistreat dingoes or dogs face severe, usually fatal, and sometimes catastrophic consequences [72]. A Ngarrindjeri (South Australia) saying decreed that boys who tickle a dingo until it “laughed” would be turned into a native-cherry tree ([140], p. 137), whilst a Mowanjum (Western Australia) myth describes how children who teasingly forced their dogs to “speak” were punished with enormous rainstorms that drowned their whole community [141]. Similarly, Wardaman people held that those who tortured dingoes were turned into pandanus palms ([142], p. 37). A tradition attributed to the Gunaikurnai (Victoria) describes an entire camp metamorphosing into stone after refusing to share their bountiful fish catch with their dingoes ([143], p. 62); another from the Karadjeri (Western Australia) describes a faithful kangaroo-hunting dingo attacking and killing his ungrateful master after repeatedly failing to receive reciprocal food or even water [144].

Physical violence towards tame dingoes and dogs, including that resulting in fatalities and occasionally their subsequent consumption, usually engendered extreme consequences, whether this was dealt with directly by the dingo or other forces. In an Arrernte (central Northern Territory) Dreaming story, for example, Ilia the emu-man teases and brutally kicks two sleeping dingoes, which immediately give chase and eventually disembowel him ([145], pp. 82–83). A group that attacked and killed another’s hunting dogs was then obliterated by extreme rain and landslides [146]. In keeping with this dingo-related eschatological theme, the killing and eating of a certain Arnhem Land man’s ‘special’ favourite dog—although unknowingly—is said to have resulted in many large camps of people being ‘wiped out’ ([147], p. 344).

Importantly, these traditions did not seek to establish that dingoes were “untouchable”: on the contrary, they were regularly disciplined for misbehaviour, and, from the beginning, dingo pups were subjected to often very rough handling by children. Yet this was considered appropriate behaviour, a key aspect of the socialisation process that was encouraged. Rather, it seems to have been wanton or purposeless cruelty—unjust abuse for human amusement, or that borne of arrogance and greed—which was considered inappropriate, with harsh penalties proscribed for such behaviour. Russell ([148], p. 181) and Kolig ([126], p. 98) record historical instances in which people who abused dingoes or dogs soon after experienced misfortunes attributed to supernatural origins, such as sickness and lightning strikes. However, such behaviour often precipitated entirely human responses as punishment. Violence amongst community members originating in disputes over the mistreatment of dingoes/dogs was commonly remarked upon ([149], pp. 32–33; [26,150,151]), as was retribution enacted upon Europeans who transgressed rules regarding their treatment ([105], p. 420; [152,153,154,155]).

It is, therefore, possible that in addition to passing down the knowledge of Dreaming events, environmental histories, and other aspects of customary “law”, Aboriginal oral traditions involving the predatory roles of dingoes served to warn members of the community about the real-world dangers these animals posed, particularly those that were wild or otherwise unfamiliar, and particularly to children. Similarly, oral traditions which described causally related punishments for unnecessary mistreatment of dingoes served to instil warnings against such actions, perhaps in order to avoid provoking potentially injurious responses from already-tamed dingoes. Collectively, they identified the moral obligations that people were responsible for following in their interactions with the dingo, both in its wild and tame forms, to successfully maintain these relationships ([156], p. 319). Indeed, after careful analysis of representations of dingoes in Aboriginal myths and oral narratives, Parker ([72], p. 195) concluded that: ‘Dingo hunting myths inform about the possible dangers of dingoes and explain ways to protect people. The myths encourage people to guard their children, to protect the old and frail and to avoid travelling or sleeping alone’. Broadly similar cautionary tales are evident in the dingo-related oral stories (“Bush yarns”) of European-Australian settler folklore prior to the early 20th century [51].

## 7. Historic and Pre-Contact Outcomes of Aboriginal Dingo Taming

In her thesis exploring the relationship of Aboriginal people and dingoes, Philip ([156], p. 92) found ‘no records of concerns about the safety of Aboriginal children and camp dingoes’, but that ‘the safety of the children and wild dingoes was taken very seriously’. Similarly, after living with isolated desert groups in the 1960s and 1970s, the anthropologist Richard Gould commented that ‘At no time did I ever see or hear of a person being bitten by [a tamed dingo]’ ([19], pp. 242–243).

In the present study, the analysis of the historical record arrived at the same conclusion. Accounts of camp dingoes harming or killing children are nearly non-existent, and, as noted, those few are so vague and difficult to verify as to be essentially apocryphal. For example, Berndt and Berndt ([147], p. 454) stated that new-born infants might be ‘gobbled up’ by dogs if the midwives’ attention focused too long on the mother, but that this was held to be ‘very rare’—on par with the frequency of babies accidentally falling out of bark cradles. The natural prey drives of tame dingoes do not appear to have been substantially suppressed, as observations of them hunting independently outside the camp for their own sustenance are so abundant as to represent the behavioural norm ([17], p. 11; [24], p. 29; [118], p. 91; [157], p. 75; [158], p. 288; [159], p. 14). Donald Thomson, for example, commented that despite keeping his pet dingo pups well-fed they still left his camp each morning to hunt in the surrounding bushland ([21], p. 17). Indeed, the same qualities that make dingoes undesirable pets in modern households presumably would have made them favoured companions in the context of a foraging lifestyle in which tamed dingoes were expected to procure game for people [14].

Nor can the apparent absence of tame dingoes’ predatory interest in Aboriginal children be attributed to a lack of appetite furnished by generous provisioning, as their relentless attempts on anything remotely edible in the camp that was not placed out of their reach became infamous amongst anthropologists. For instance, at some field camps, tame dingoes kept by Indigenous informants pilfered and ate everything from the researchers’ tinned food to their toothpaste ([74], p. 15), boots, magazines, electrical cables, and in one case an entire box of detergent ([18], p. 65) (see also [20]; [25], p. 290; [160], p. 7; [161]; [162], p. 109). Aboriginal people accommodated this aspect of their tamed dingoes’ behaviour in various ways, such as by keeping leftover food on the tops of domestic shelters ([19], p. 20), or on purpose-made storage platforms ([74], p. 50; [82]). Special provisions were also made for dingo-scavenging in mortuary customs: in southeastern Victoria, for instance, graves were covered with logs ‘to keep the dingoes out’ ([163], p. 466). It does seem, however, that despite the incessant hunger and irrepressible prey drive of camp dingoes, these animals seemed to comprehend that infants and children were not only not to be preyed on—they were not to be harmed in any way.

As has been previously indicated, Aboriginal people did perceive *wild* dingoes as serious threats to humans, although typically only children, and particularly infants; indeed, as already noted there is some evidence, albeit rather slender, for this eventuating as an issue. By contrast, one early observer praised the protective nature of Victorian-tamed dingoes:

They were usually bred in a domesticated state, and no puppies were ever destroyed. Wild young ones were also caught and domesticated. The dogs were trained to guard the wuurns [dwellings], which they did by growling and snarling … In watching they were vigilant and fierce. They would fly at the throats of visitors; and strangers had often to take refuge from them by climbing into a tree.

([118], p. 89)

Successfully tamed, not only were dingoes no longer regarded as a threat to members of their own community but were actively valued for their contributions to security against outsiders—including malevolent spiritual forces that are normally invisible to humans ([19], p. 23). Indeed, there are Aboriginal accounts of tame dingoes being entrusted to guard children whilst their parents were out foraging [141]. An early chronicler of Aboriginal life documented a possible example of this “guardianship” near Port Stephens in coastal New South Wales, coming across a daytime camp inhabited only by a small girl who was ‘sleeping soundly between two dogs’ ([23], p. 245)—given the early context these could have been tamed dingoes, although it is not certain [16]. An early European explorer of the New South Wales interior, Thomas Mitchell, also recounted the resolve of a tame dingo to protect two young Aboriginal children, one of whom was blind, who his party encountered alone in a camp on the Lachlan River in 1836:

A large supply of the balyan root lay near them, and a dog so lean as scarcely to be able to stand, drew his feeble body close up beside the two children as if desirous to defend them. They formed indeed a miserable group, exhibiting nevertheless instances of affection and fidelity creditable both to the human and canine species.

([107], p. 60)

It is not just the risk of a tame dingo carrying off and eating a baby or small child that could have been a factor in the Aboriginal–dingo relationship. Living with a tamed wild predator of this kind in a domestic setting clearly involves significant management issues. There are obviously plentiful examples from recorded history and the contemporary world of people keeping dangerous animals like big cats as household pets that from an early age have been tamed and habituated to their owners and wider adoptive family (including pets of other species), and hence do not regard them as “food” [164,165]. Due to their large size and formidable strength and power, however, even immature big cats can still easily kill or severely injure human adults through mild antagonistic behaviours or a “playful” bite or paw swipe [166]. A tame dingo would presumably be less of a threat in this regard than, say, a tiger, owing to its much smaller size. However, the play behaviour that dingoes use to establish pecking order amongst youngsters often involves forcing the “loser” into a grounded position with their vulnerable spots (neck/belly) exposed, and if they seriously want to hurt they tend to attack the neck area [1]. This sort of dominance behaviour by a tame dingo certainly could/would cause grievous injury to a child. It is therefore noteworthy that tame dingoes were apparently not considered a threat to the safety of Aboriginal children.

Early European writers frequently opined that Aboriginal people valued their camp dingoes as highly as their own children, if not more; would mourn the loss of a tamed dingo with more profound grief than that of a child, and so on (e.g., [88,99,155,167,168,169]). Such a notion, if correct, could imply that the death of an Indigenous child through an attack by a tamed dingo—whether predatory in motivation or related to “play” behaviour—might have been accepted because the benefits of dingo-keeping outweighed the value of the life of an individual child. It seems worth noting, for example, that Oromo people in Ethiopia tolerate occasional attacks on community members (including predation of children) by the wild hyenas that live near their settlements because it is believed they confer protection from evil spirits [170]. It is more likely, however, that inferences found in the early literature that tamed dingoes were worth more to Aboriginal people than their own children were grounded in the many misconceptions about Indigenous society that took root in colonial folklore. They were no doubt also influenced by the colonialist mindset that Aboriginal morality was innately inferior to Europeans’ [171].

### Possible Archaeological Evidence for Long-Effects of Dingo Taming

It is important to note that the archaeological record indicates an additional and highly significant behavioural modification resulting from the Aboriginal taming of dingoes: the suppression of their innate tendency to revert permanently to the wild upon sexual maturity. Skeletal remains of dingoes are not uncommonly found in archaeological sites from southeastern Australia, and the better-preserved instances are typically the remains of tamed individuals that were deliberately buried within the site. Several of these, mostly believed to date to within the last 1000 years, were examined in Gollan’s osteological assessment of prehistoric dingo “fossil” material [172,173]. In these studies he produced estimates of the ancient dingoes’ age at the time of death based on relative dental wear, the closure of cranial sutures, development of cranial cresting and fusion of postcranial limb bone epiphyses [172,173]. Gollan’s analyses confirmed that these specimens reflected a mixture of males and females and that many of these individuals had long been sexually mature (Table 1). The specific patterning of their dental attrition—consistently different to that of modern wild dingoes—also suggested that these tamed dingoes had been throughout the course of their lives reliant on human provisioning, rather than independent capture and processing of large game ([173], p. 341).

With this evidence for the longer-term residence of tamed dingoes within Aboriginal camps established, the natural next question is whether these individuals were also reproducing within the domestic environment. One of Gollan’s [172] main findings was that some pre-contact tamed dingoes, specifically those from southern coastal and western riparian bioregions of New South Wales, exhibited morphological traits reminiscent of domestication such as reduced overall size, crowded and diminutive dentition, and altered cranial proportions giving a gracility of shape. Together, these present a drastic departure from the phenotype dominant in wild dingoes that Gollan [172] interpreted as the product of tamed individuals breeding within the camps with one another, effectively in isolation from the broader wild population.

On this note, the remains of new-born dingoes have been found in some archaeological sites, but these await specialised study. Of particular interest to this discussion are cases such as Currarong (on the New South Wales South Coast [Figure 2]) where their remains are not burned or otherwise modified and much of the skeleton is present together (e.g., [174]), suggesting that they are not likely to constitute wild dingo pups that were eaten, as described historically [33,34]. Rather, they quite possibly represent offspring born to tame dingoes residing within the camp that did not survive past a young age; under the typical nutritional stress described earlier, the rate of mortality amongst camp-born litters was likely to have been high [22] and indeed is high in more recent camp dogs [83]. However, it is presently impossible to rule out that these were wild-born pups that simply died very soon after being brought to camp from malnutrition or disease, maltreatment (e.g., at the hands of children), or intentional culling.

## 8. Discussion and Conclusions

The wild dingo socialisation method employed by Indigenous foragers in mainland Australia seems to have been a unique process that is not yet fully understood, and which appears to have no apparent real parallels in other recent or historically-attested traditional societies. Numerous examples are known from the ethnographic record of foraging groups or other small-scale human societies capturing juvenile animals in the wild and hand-rearing and taming them as companions (often involving cross-species wet-nursing), and, in some instances, the socialised animals did eventually return to the wild [113,165]. It would appear to be the case, however, that the Aboriginal–dingo relationship examined here is the only documented example of such a special bond arising between humans and a wild canid species. Some small-scale societies in the northern hemisphere hand-reared the captive wild-born pups of grey wolves (*C. lupus*); however, it was typically the case that these communities kept and tamed the juvenile wolves with the intention of killing them for consumption or ritual purposes, rather than allowing them to revert to the wild [40,41].

Taken together, the historical and archaeological evidence indicates that the socialisation process Aboriginal people employed in taming dingoes produced individuals with modified behavioural tendencies. The natural predatory instincts of these captive wild carnivores were somehow suppressed in regard to young humans, and in some areas, they appear to have been content to remain in the camp instead of dispersing upon attaining sexual maturity. Some of these older sexually and physically mature camp dingoes likely reproduced in the boundaries of human foraging settlements or at least within their vicinity with other tamed dingoes [32]. Where this occurred over extended periods of time, perhaps facilitated by the economic lifeways, higher population density, and more sedentary nature of Aboriginal peoples of southeastern Australia, it seems to have also produced visible phenotypic modifications [33,34]. However, it would appear that any longer-term processes of dingo management were interrupted—permanently—by the disruptions to traditional Aboriginal lifeways caused by European colonisation in 1788. Regardless, it is clear that the various successes of Aboriginal efforts to tame dingoes stand in stark opposition to more recent European efforts to tame and keep dingoes in captivity and to generally raise and treat them “as dogs”.

### Implications for the Domestication of the Wolf

The Aboriginal–dingo relationship as presently conceived has implications for the current understanding of the original domestication of dogs from the Eurasian grey wolf—or an extinct sub-population(s) of these wild canids, as is presently believed to have been the case [175]. Current evidence suggests the earliest domesticated dogs can be traced back to 16–14 thousand years (ka) ago in the archaeological record [176,177], and more contentiously up to ~40–30 ka ago on the basis of genetic data [178] and morphological analyses [40,41,179]. There are two competing hypotheses that purport to explain the beginnings of wolf domestication [40,41,42,43]. The self-domestication or commensal scavenger model [43] posits that some wild wolves acquired the habit of scavenging carrion and edible waste generated by mobile communities of Late Pleistocene foragers; the wolves that lived commensally with humans then diverged slowly from the wider free-ranging population as they adapted genetically to this novel ecological niche [114].

The alternative hypothesis for initial dog domestication—and one that is increasingly gaining traction (but see [31])—is the cross-species adoption or pet-keeping model [40,41,42,43]. The basic premise is that Late Pleistocene hunter-gatherers in Eurasia had a longstanding cultural practice of taking pre-weaned wolf pups (~8–10 days old) from wild dens and hand-rearing the more sociable and playful of these captive individuals as companions (i.e., pets) [40,41,42,43]. Those pups that were unable to adapt to life in captivity were culled or escaped into the wild. The adopted young animals became closely bonded to their human caregivers through an intensive phase of nurturing and socialisation in which cross-species wet nursing was central [40,42]. Importantly, it is argued that the wild-caught pups with “friendlier” dispositions would have been retained as pets beyond sexual maturity. These animals yielded one or more litters of pups that became the property of humans [40]. Human selection pressure then operated on successive generations of this breeding stock [40]. It is surmised that generation after generation of socialised adult wolves reproducing in or close to hunter-gatherer communities, coupled with continuous human selection for behavioural tameness in each new litter of pups, gave rise to an evolutionarily divergent lineage of canids—the first dogs [41].

Critics of the pet-keeping model, on the other hand, argue that it is unrealistic to infer that adopting wild-born wolf pups as family pets could ever result in domestication because modern experience suggests that socialised wolves are highly undesirable companions [114]. Such perceptions are essentially based on the results of wolf socialisation experiments (e.g., [180,181]). Wolves that are hand-raised in these programs can become closely bonded to their individual human caregivers [182]. They invariably become more difficult to manage, however, as they grow in size and strength, and especially when they are sexually mature [114]. Modern experience shows that individualised attachment bonds can be maintained with socialised adult wolves [181,183]. However, these genetically-wild animals still have a strong prey drive and are prone to subjecting humans to dominance tests that can quickly escalate into serious attacks [184]. They also have a predilection to attack people displaying any form of debility, including their primary carers [184]. Additionally, their predatory motor patterns are often triggered by the noises and movements of small children; indeed, contact between socialised adult wolves and human youngsters is scrupulously avoided by handlers ([184], p. 53). In short, most socialised adult wolves are formidable animals that are kept in secure enclosures and handled with care for good reason [184].

Clearly, there is much here that resonates with the human–dingo relationship. In light of the findings of the present review, it can be contended that if the pet-keeping theory [40,41,42,43] does provide a valid model for how dogs were initially domesticated from wolves then it is probably only because foragers were somehow able to resolve the perennial problem of how to socialise young wild canids to live in the domestic space whilst trying to minimise the danger posed to the human residents of those communities, and to infants and small children in particular; and also while working to keep them around in the long term such that reproduction in or near the camp occurred.

This may have been accomplished by forging a relationship with juvenile wolves taken from the wild that was similar to that between Aboriginal people and captive dingoes in Australia; in particular: (1) hand-rearing and intensively socialising wild-caught wolf pups together with human babies and children; (2) encouraging rough-handling of (and/or rough “play” with) adopted wolf pups by youngsters; and (3) notwithstanding the former, inculcating young people to treat tame wolves with care and respect—lessons imparted through formal instruction and communicated in oral tradition (i.e., myths and other forms of storytelling) and lore.

Owing to the larger size of wolves it probably would have been necessary for Late Pleistocene Eurasian foragers to be more severe about culling captive wolf pups that persistently responded to this treatment with defensive aggression (i.e., kill or abandon those that could not be habituated to rough-handling by children), thus actively selecting for docility and other people-friendly traits [33,34]. On the other hand, it is possible that the nearest ancestor of the dog was from eastern Asia, where the wolves are generally smaller and less powerful than *C. lupus* in western Eurasia ([185,186,187], see also [33,34,175]).

A tradition of human–wolf companionship that was similar to the Aboriginal–dingo association could have had the effect of suppressing the predatory behaviour of wild-caught wolf pups around small children; making it possible for foragers to reside safely with these animals in the domestic space for progressively longer periods of time after they had matured sexually and physically. It would have been important for this relationship to endure without disruption in one area over multiple generations. It may then have been possible for genetic changes associated with human selection to have coalesced to the extent that canids were being born in or near foraging communities that were distinct from solely wild-living wolves from both morphological and behavioural perspectives—an early stage in the domestication process.

Also likely to be important was the establishment of a commensal wolf population comprised of captive-reared wolves that had dispersed to the wild to breed. This would mean that each birthing season the pups foragers acquired from local dens were often the progeny of wolves that had been subjected to an intergenerational process of human selection ([33,34], see also [32]). In addition, human-induced genetic changes are more likely to have occurred if the wolf population was situated in a glacial refugium or other biogeographical contexts that brought wolves and humans closer together, isolated the former from a reproductive standpoint, and kept the wolves subject to human selection pressures. As dogs first appear in the archaeological record during and immediately after the Last Glacial Maximum (26.5–19 ka ago [188])—and seem to derive from East Asian wolves somewhere south of Siberia proper [175]—it is likely that this process occurred within the vicinity of modern-day China. The earliest dogs could possibly have arisen from the practice of adopting pups from a wolf population with such a uniquely close human association.

## Figures and Tables

**Table 1:** Demographic information of archaeological tamed dingoes as reported by Gollan [172]. Sex was mostly determined using the presence/absence of a baculum. Where bones of the pelvic area were not preserved, via metric and non-metric dimorphic traits; some of the latter identifications are therefore best estimates. Highly incomplete specimens for which reasonable assessment was impossible were omitted. Locations of sites are shown in Figure 2.

Archaeological Site	Specimen ID	Estimated Age	Sex
CCLP Kurnell, NSW	BB4/F4	>1.5 years	Est. F
CCLP Kurnell, NSW	BB4/G5B	>1.5 years	Est. M
Nundera Point, NSW	ANU Kioloa 01	>1.5 years	M
WOC-1 Lake Mungo, NSW	ANU Mu 01	Adult	Est. F
Lake Milkengay, NSW	ANU Milk 01	>1.5 years	Est. F
Murramarang, NSW	SHELL Mur 01	~6 months	F
Murramarang, NSW	ANU Mur 02	>1.5 years	M
CSP-1 Mallacoota, VIC	ANU Mal 01	>4 years	M
CSP-1 Mallacoota, VIC	ANU Mal 02	~5 months	M
Fromm’s Landing, SA	SAM Fromm 01	~5–6 months	M

## Data Availability

Not applicable.
